# Birth Patterns in the Aftermath of the 1918 Influenza Pandemic in India: The Case of Madras City

**DOI:** 10.1111/irv.13355

**Published:** 2024-07-25

**Authors:** Siddharth Chandra, Rajiv Sarkar, Banseilang Rynjah

**Affiliations:** ^1^ Asian Studies Center, James Madison College, and (by courtesy) Department of Epidemiology and Biostatistics Michigan State University East Lansing Michigan USA; ^2^ Indian Institute of Public Health Shillong Shillong Meghalaya India

**Keywords:** 1918 influenza pandemic, births, deaths, India, Madras, time series analysis

## Abstract

This paper examines the timing of one‐time fluctuations in births subsequent to the 1918 influenza pandemic in Madras (now Chennai), India. After seasonally decomposing key demographic aggregates, we identified abrupt one‐time fluctuations in excess births, deaths, and infant deaths. We found a contemporaneous spike in excess deaths and infant deaths and a 40‐week lag between the spike in deaths and a subsequent deficit in births. The results suggest that India experienced the same kind of short‐term postpandemic “baby bust” that was observed in the United States and other countries. Identifying the mechanisms underlying this widespread phenomenon remains an open question and an important topic for future research.

## 1 Introduction

With a population of over 1.4 billion and, since April 2023, the most populous country in the world [1], India has attracted great interest from demographers and epidemiologists alike since it gained independence in 1947 [2]. India was the single worst affected country during the 1918 influenza pandemic, with an estimated loss of life of 18 million people in the directly ruled provinces in the short span of a year [3]. Despite this, research on the 1918 influenza pandemic in India is surprisingly thin compared with research on the United States and other high‐income countries [4, 5, 6, 7, 8]. Furthermore, very little is known about the demographic sequelae of the pandemic in India and the questions that they raise about the disease.

While there is literature on birth outcomes in the aftermath of the 1918 pandemic, the focus is mostly on countries comprising the “global north.” A large one‐time decline in births 9–10 months after the October 1918 peak of the pandemic has been reported in the United States [9], Japan [10], and Taiwan [11]. Khare et al. reported a peak in stillbirths approximately 9–10 months after the peak in mortality during the 1918 influenza pandemic in the United States [12]. Contrary to the popular belief that fertility increases after a disaster, a recently published systematic review reported an overall negative short‐term effect of disasters on fertility [13]. Over the slightly longer term, Mamelund found that fertility increased in Scandinavia, following an initial decrease immediately after the pandemic [14], although this phenomenon has been attributed to the aftermath of the First World War rather than the waning of the pandemic [15].

This study utilizes data from the city of Madras (presently Chennai) in India to determine (i) whether there is a link between pandemic‐associated excess deaths and subsequent births and (ii) to evaluate the length of the time lag between changes in excess deaths and future births. According to the Sanitary Commissioner's report, the maximum death toll in Madras exceeded 200 deaths per week between June and December 1918 [16]. An estimated 30% reduction in births in India was noted in the year 1919, most likely due to the severity of the pandemic [4].

**FIGURE 1:**
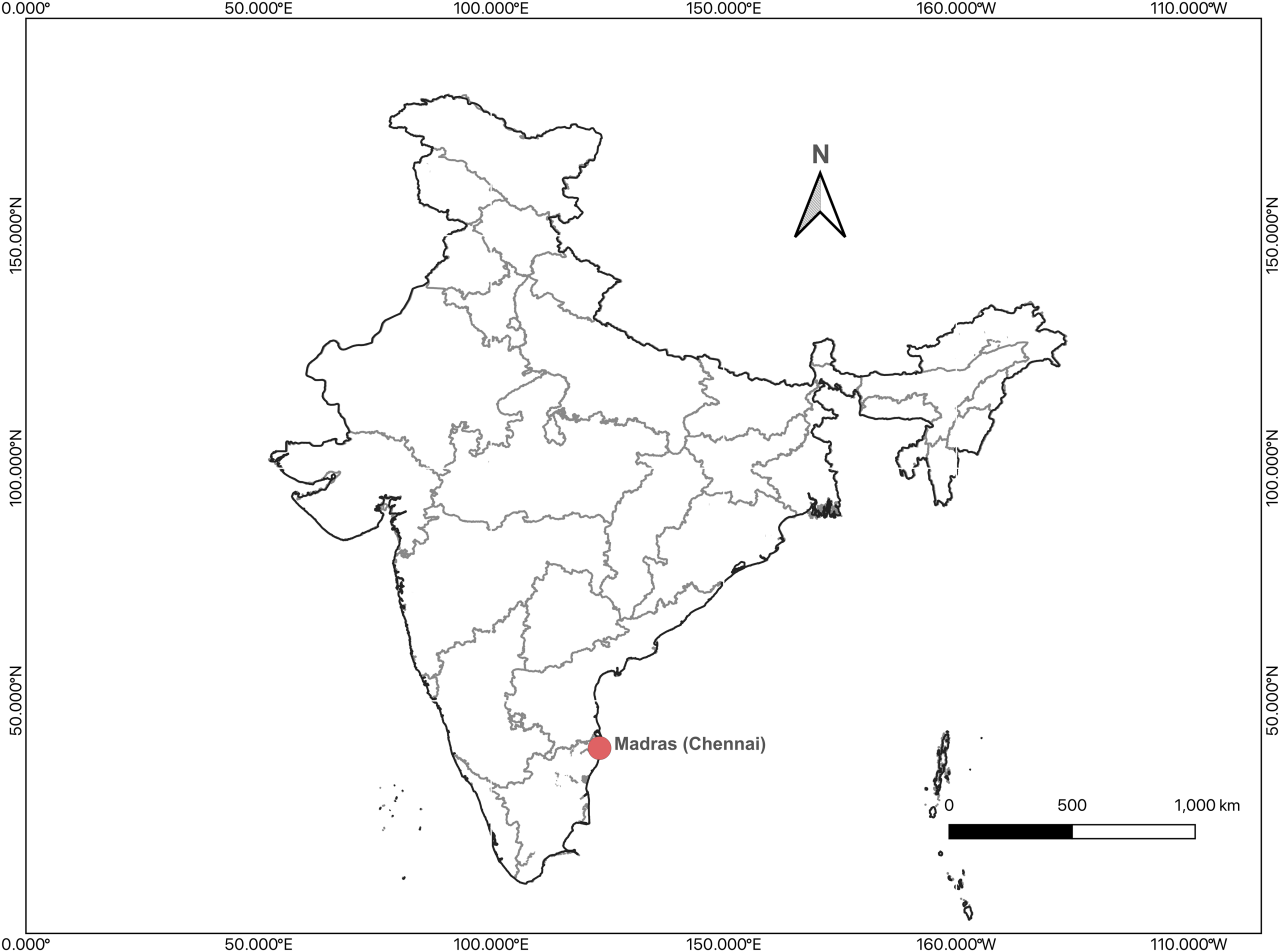
Map of India showing the location of Madras (present Chennai).

Chennai is the capital of Tamil Nadu, the southernmost state of India. Located on the Coromandel coast off the Bay of Bengal (Figure 1), it enjoys a tropical climate with average temperatures ranging from 25°C in January to 32°C in May [17]. The city receives most of its annual rainfall (average 1250 mm) between October and December [17].

## 2 Data and Methods

The data for this study included monthly counts of deaths, births, and infant deaths from 1914 to 1923 in Madras City, published by the Corporation of Madras (Madras Health Department, 1914–1923) [18]. These data enabled us to analyze the magnitude of and temporal association between monthly deaths, births, and infant deaths during the 1918 influenza pandemic.

We used time series techniques to perform our analysis using SAS. Using the PROC X12 procedure, we additively decomposed each of the three time series (births, deaths, and infant deaths) into three separate components: seasonal (cyclical), trend, and irregular (random). The cyclical component captures systematic seasonal fluctuations that are known to occur in key demographic aggregates such as births and deaths. The trend component captures longer term trends in the time series—in a growing population, for example, one may expect to see an exponential growth trend in births, which is a common feature of population growth models. The irregular (random) component is the difference between the original series and the sum of the seasonal and trend components. After decomposing the series, we extracted the irregular (random) component of each series for further analysis. This irregular component can be interpreted as the excess or deficit in births, deaths, or infant deaths after adjusting for seasonal and trend factors. To the extent that an anomalous event like the 1918 influenza pandemic would have had an impact on key demographic aggregates, we would expect to see those impacts in the irregular components of the birth, death, and infant death data.

After extracting the irregular components of the monthly birth, death, and infant deaths, we interpolated the time series to produce weekly estimates of excess births, deaths, and infant deaths. Finally, we computed two sets of pairwise cross‐correlations, (i) between deaths and births and (ii) between deaths and infant deaths, at different time lag and lead intervals, to determine the temporal associations among these key demographic aggregates.

## 3 Results

**FIGURE 2:**
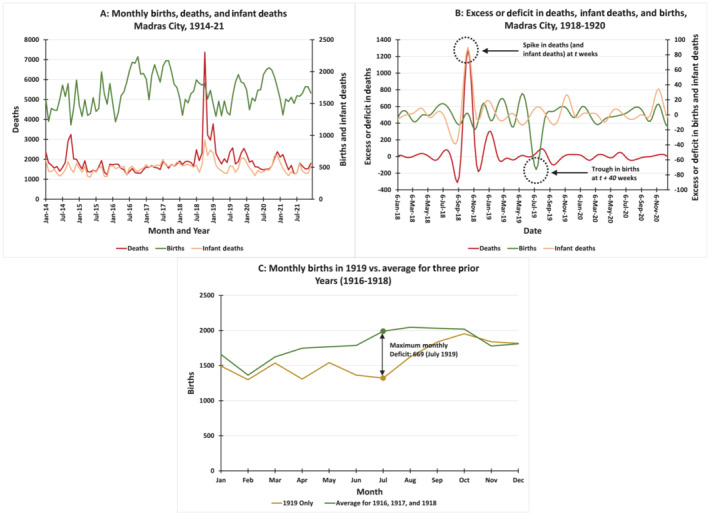
(A) Monthly births, deaths, and infant deaths, 1914–1921 (top left). (B) Excess or deficit births, deaths, and infant deaths, 1918–1920 (top right). (C) Monthly birth deficit in 1919 relative to 1916–1918 average (bottom).

Figure 2 shows the plots of the original monthly birth, death, and infant death counts (Figure 2A, left). The most pronounced feature of this figure is the spike in deaths and infant deaths in October 1918, corresponding to the devastating pattern of high mortality during the “Fall Wave” of the pandemic. Notably, the pattern of births subsequent to this wave does not appear to be abnormal. However, when seasonal and trend factors are filtered out to reveal the excess or deficit in patterns of births (Figure 2B, right) and those data are interpolated to yield a weekly series, we see a deep trough in excess births approximately 40 weeks (~9–10 months) after the peak in excess deaths and infant deaths. These observations visually support the notion of a lag corresponding to the length of human gestation between the peak of the pandemic, measured by deaths or infant deaths, and the trough in births.

Next, we estimated the magnitude of the deficit in births by subtracting the births for each month between April and October 1919 from the corresponding means for the years 1916–1918 (Figure 2C). The birth deficit during these 6 months totaled 2372, or 20.87% of the normal number of births. For July 1919, the deficit of 669 was 33.62% of the normal level, and for the entire year 1919, which included a few “normal” months, the deficit was 12.43% of the normal level.

**FIGURE 3:**
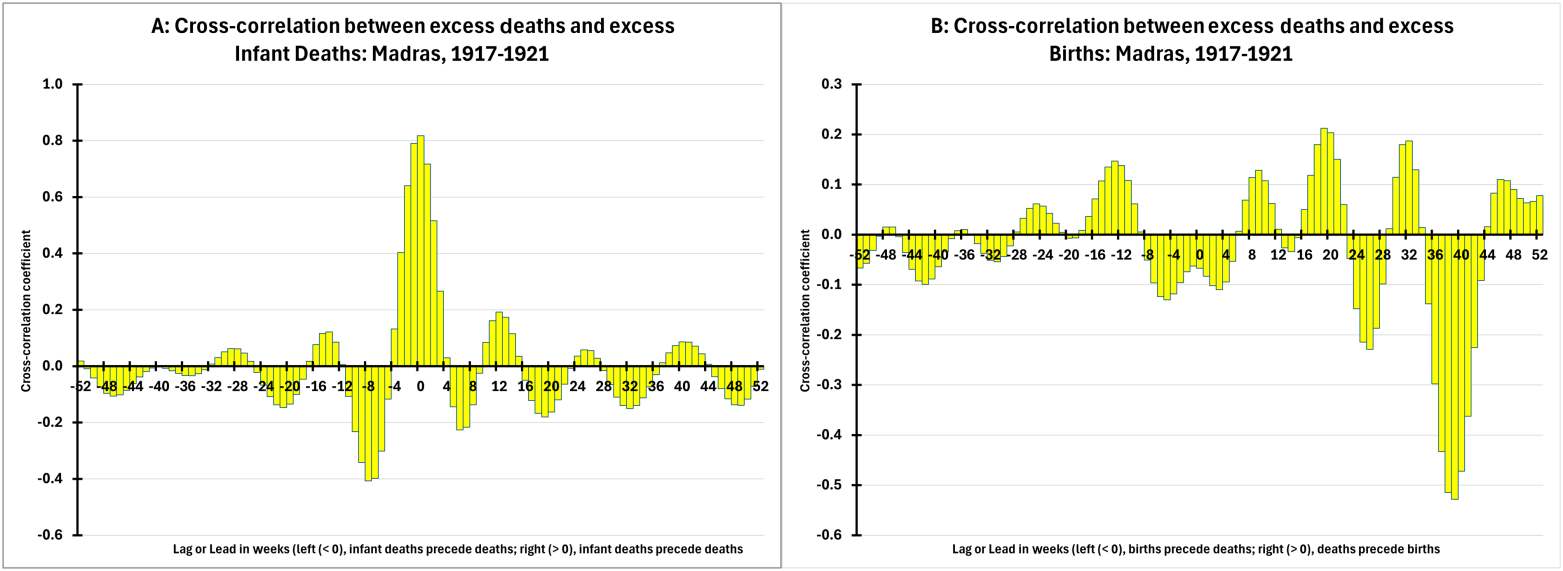
Cross‐correlations for excess deaths, infant deaths, and births: Madras, 1917–1921.

Finally, we computed two cross‐correlation functions to test and verify our observation of the 40‐week lag between excess deaths and the deficit in births. Figure 3A shows the cross‐correlations between excess deaths and excess infant deaths, and Figure 3B shows the cross‐correlations between excess deaths and excess births at *k*‐week lags or leads (−52 ≤ *k* ≤ 52). Figure 3A shows a positive and statistically significant correlation between deaths at time *t* and contemporaneous infant deaths (i.e., also at time *t*; (0) = 0.818)). This suggests, unsurprisingly, that excess deaths and infant deaths tend to fluctuate in tandem. Of greater interest is Figure 3B, which shows a large and negative correlation between deaths and births 40 weeks later (i.e., time *t* + 40; (40) = −0.528)). Coupled with Figure 2B, this finding confirms the fact that excess deaths at the peak of the 1918 pandemic predict birth deficits with a lag equal to the average gestation time for humans.

## 4 Discussion and Conclusion

Prior research focusing on a variety of locations in the “global north” has demonstrated a 9–10 month lag between peak mortality during the 1918 influenza pandemic and a steep decline in births. However, this important demographic phenomenon has not been demonstrated for India, the most populous country in the world and the country that was the worst affected by the pandemic, due to the lack of country‐wide monthly data on births. The discovery of such data for a major Indian city, Madras, enables the analysis of this phenomenon in the context of India.

The results of this study confirm that Madras (and, possibly, other locations in India) experienced a dip in births with a 40‐week lag, representing the duration of human gestation. This finding suggests that the short‐term “baby bust” was more widespread globally than previously known. It also raises the question about why the experiences of so many countries across the globe were so similar. While the baby bust, resulting from a failure to conceive, seems to be closely connected to the severity of the pandemic, failure to conceive itself can result from biological and/or behavioral mechanisms [9]. Identifying the specific mechanisms which led to this widespread phenomenon remains an open question and an important topic for future research and should be of interest to researchers studying demographic trends in the aftermath of the recent COVID‐19 pandemic.

## Author Contributions

**Siddharth Chandra:** conceptualization, methodology, writing – review and editing, writing – original draft, formal analysis, funding acquisition, visualization, software, investigation, data curation, supervision. **Rajiv Sarkar:** conceptualization, methodology, investigation, writing – review and editing, writing – original draft, supervision. **Banseilang Rynjah:** formal analysis, writing – original draft, data curation.

## Conflicts of Interest

The authors declare no conflicts of interest.

## Data Availability

The reports from which the data that support the findings of this study were derived are available from the Wellcome Collection (https://wellcomecollection.org/). These data were derived from the annual *Report of the Health Officer, Corporation of Madras Health Department*, published in various years. For example, the data for 1917 are available at https://wellcomecollection.org/works/dt58vkan, and the following search will yield all the reports from which data for this study were drawn: https://wellcomecollection.org/search/works?query=Madras+%28India%29.+Health+Department.
